# Complete Plastome Sequence of *Grimmia tergestina* Provides a Genomic Resource for Grimmiaceae

**DOI:** 10.3390/genes17050572

**Published:** 2026-05-18

**Authors:** Hengyu Dai, Shouqiang Li, Huakun Zhou, Xiaojuan Li, Jiuli Wang

**Affiliations:** 1 College of Ecological Environment and Resources, Qinghai Minzu University, Xining 810007, China; daihengyu2020@163.com (H.D.); 15719701919@163.com (S.L.); 2 Qinghai Provincial Key Laboratory of Restoration Ecology in Cold Region, Northwest Institute of Plateau Biology, Chinese Academy of Sciences, Xining 810007, China; hkzhou@nwipb.cas.cn; 3 Qinghai Provincial Key Laboratory of High-Value Utilization of Characteristic Economic Plants, Xining 810007, China; 4 Qinghai Provincial Biotechnology and Analytical Test Key Laboratory, Qinghai Minzu University, Xining 810007, China

**Keywords:** *Grimmia tergestina*, chloroplast genome, plastome, Grimmiaceae, cpSSR, phylogenetic placement, bryophytes

## Abstract

Background/Objectives: *Grimmia tergestina* is a lithophytic moss of Grimmiaceae, but its complete plastome has not previously been reported. This Brief Report presents the complete chloroplast genome of *G. tergestina* as a genomic resource for future work on species identification, phylogeny, and plastome evolution in Grimmiaceae. Methods: Illumina NovaSeq PE150 reads were quality filtered and assembled into a circular plastome. Genome annotation was verified using current organellar annotation tools and manual curation, and a preliminary phylogenetic analysis was performed using shared chloroplast protein-coding genes from representative moss plastomes. Results: The complete plastome of *G. tergestina* was 124,153 bp in length and exhibited the typical quadripartite structure of moss plastomes. It encoded 126 genes, including 82 protein-coding genes, 36 tRNA genes, and 8 rRNA genes, with an overall GC content of 28.49%. Fourteen genes contained introns, and nine genes were duplicated in the inverted repeat regions. Codon-usage analyses showed a preference for A/U-ending codons, consistent with the AT-rich composition of the plastome, and supplementary ENC and PR2 analyses supported a conservative interpretation of codon-usage bias. A total of 569 chloroplast simple sequence repeats and 222 dispersed repeats were identified. The preliminary maximum-likelihood phylogeny placed *G. tergestina* within Grimmiaceae and resolved it close to *Niphotrichum japonicum* in the sampled plastome dataset. Conclusions: The newly characterized plastome of *G. tergestina* enriches genomic resources for *Grimmia* and provides a foundation for future comparative and phylogenetic studies of Grimmiaceae.

## 1. Introduction

Bryophytes represent one of the earliest-diverging lineages of land plants and provide important information for understanding the diversification of terrestrial plants. In recent years, complete plastome sequences have become useful genomic resources for bryophyte systematics, species identification, and comparative studies because they provide conserved gene order together with lineage-informative sequence variation [1,2].

*G. tergestina* Tomm. ex Bruch & Schimp. belongs to Grimmiaceae and is a lithophytic moss that commonly occurs on exposed rock surfaces. Species of *Grimmia* are taxonomically important in Grimmiaceae and include many taxa associated with rocky or dry habitats [3,4]. However, genomic resources for this genus remain limited, and complete plastome information is still unavailable for *G. tergestina*.

Previous moss plastome studies have shown that many bryophyte chloroplast genomes are structurally conserved, with a typical quadripartite structure composed of a large single-copy region, a small single-copy region, and two inverted repeat regions [5,6]. Therefore, for species represented by a single newly sequenced plastome, the most appropriate contribution is often the release of a reliable genomic resource rather than broad claims about adaptive evolution. In this context, the plastome of *G. tergestina* can provide a useful reference for future studies of Grimmiaceae and related mosses.

In this study, we assembled and annotated the complete plastome of *G. tergestina* and summarized its basic genomic features, repeat composition, codon-usage pattern, nucleotide diversity, and preliminary phylogenetic placement among representative moss plastomes.

## 2. Materials and Methods

### 2.1. Plant Material, DNA Extraction, and Sequencing

Fresh gametophyte material of *G. tergestina* was collected in October 2024 from alpine rocks on the southern bank of the Yellow River in the Huangheqing National Wetland Park, Guide County, Hainan Tibetan Autonomous Prefecture, Qinghai Province, China (36.051258° N, 101.301659° E; altitude: 2190.68 m). A voucher specimen was deposited in the Sample Room of the Qinghai Provincial Biotechnology and Analytical Test Key Laboratory, Qinghai Minzu University, Xining, China, under voucher number HHQ2024001.

Clean gametophyte tissues were frozen in liquid nitrogen and transported on dry ice. Total genomic DNA was extracted using a Universal Plant DNA Extraction Kit (Nanjing Jisihuiyuan Biotechnology Co., Ltd., Nanjing, China, D312) following the manufacturer’s protocol. DNA concentration and purity were evaluated using a One Drop OD-1000+ ultramicro spectrophotometer (One Drop Technologies, Nanjing, China), and DNA integrity was assessed by 1% agarose gel electrophoresis at 120 V for 25 min using a DL2000 DNA Marker (TSJ011-100; Tsingke Biotechnology Co., Ltd., Beijing, China). The DNA concentration was 10.94 ng/μL, with a total amount of 0.44 μg, and the sample was qualified for subsequent library construction. Paired-end libraries were constructed after DNA fragmentation by ultrasonication, fragment purification, end repair, 3′-end adenylation, adaptor ligation, size selection by agarose gel electrophoresis, and PCR amplification. Qualified libraries were sequenced on the Illumina NovaSeq 6000 platform (Illumina, San Diego, CA, USA) in PE150 mode by Nanjing Jisihuiyuan Biotechnology Co., Ltd., China. Raw reads were filtered using fastp v0.23.4 [7] to remove adaptor sequences, primer sequences, reads with an average quality score lower than Q5, and reads containing more than five ambiguous bases. After filtering, 29,346,982 clean paired-end read pairs comprising 8,862,788,564 bp were obtained, with Q20 and Q30 values of 98.30% and 93.77%, respectively.

### 2.2. Plastome Assembly, Validation, and Annotation

Chloroplast reads were extracted by mapping the clean reads against an in-house chloroplast genome database using Bowtie2 v2.2.4 [8] in very-sensitive-local mode. The plastome was assembled de novo using GetOrganelle v1.7.7.1 [9], with k-mer values of 55, 87, and 121. Assembly quality was assessed by mapping reads back to the assembled plastome and by comparing the assembled sequence with a reference plastome. The final plastome was recovered as a circular molecule with an average coverage depth of 272.98×. A total of 109,407 paired-end reads mapped back to the assembled plastome, and the estimated insert size was 329.28 ± 70.46 bp. The assembled plastome was deposited in the China National GeneBank under accession number CNS1408476 and in GenBank under accession number PZ382285.

To address current standards for organellar genome annotation, the plastome annotation was verified using GeSeq and CPGAVAS2 online tools, accessed on 1 February 2026 with closely related moss plastomes as references [10,11]. Transfer RNA genes were checked using tRNAscan-SE online tools, accessed on 3 May 2026 [12]. Gene boundaries, start and stop codons, and intron-containing genes were manually inspected. A circular plastome map was generated using OGDRAW [13].

### 2.3. Genome Feature, Repeat, Codon Usage, Nucleotide Diversity, and IR Boundary Analyses

Basic plastome features, including genome size, GC content, gene content, and intron-containing genes, were summarized from the final annotation. Chloroplast simple sequence repeats (cpSSRs) were identified using MISA-web online tool, accessed on March 2026, with thresholds of 8, 5, 3, 3, 3, and 3 repeat units for mono-, di-, tri-, tetra-, penta-, and hexanucleotide motifs, respectively [14]. Dispersed repeats were identified using vmatch v2.3.0 with a minimum repeat length of 30 bp and a Hamming distance of 3 [15]. Relative synonymous codon usage (RSCU) was calculated from high-quality protein-coding sequences longer than 300 bp, beginning with a standard start codon, ending with a standard stop codon, and lacking internal stop codons [16]. To evaluate codon-usage bias more cautiously, effective number of codons (ENC), GC3s, and parity rule 2 (PR2) analyses were also performed [17,18]. Codon-usage analysis was performed to provide a descriptive overview of the codon-usage pattern in the plastome. Complete plastome sequences were aligned using MAFFT v7, and nucleotide diversity (π) was calculated in DnaSP v6 using a fixed-size sliding-window approach. Following parameter settings commonly used in plastome comparative studies, the window length and step size were set to 1000 bp and 500 bp, respectively [19]. This analysis was used only to identify relatively variable regions for future marker development and was not used to infer adaptive divergence. LSC/IR/SSC junctions were compared among representative moss plastomes using the IRscope online program, accessed on 3 March 2026 [20].

### 2.4. Phylogenetic Analysis

To provide a preliminary phylogenetic placement of *G. tergestina*, representative moss plastomes were retrieved from public databases, including Grimmiaceae and related Dicranidae taxa where available, with *Sphagnum* species used as outgroups. The taxa and chloroplast genome accessions used for phylogenetic analysis are listed in Table S1. Shared chloroplast protein-coding genes were extracted, aligned using MAFFT v7 [21], concatenated, and analyzed under the maximum-likelihood framework in MEGA 12 with 1000 bootstrap replicates [22]. Because currently available complete plastomes for Grimmiaceae remain limited, the tree was used for preliminary placement rather than for a comprehensive reconstruction of relationships within the family.

## 3. Results

### 3.1. General Features and Gene Content of the Plastome

**Figure 1:**
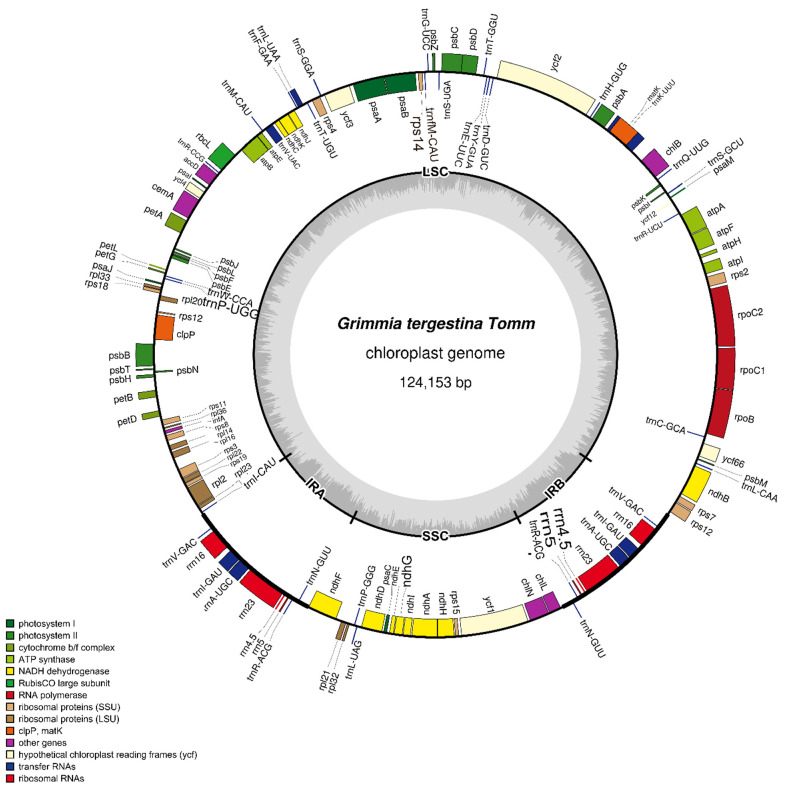
Circular map of the chloroplast genome of *Grimmia tergestina*.

The complete plastome of *G. tergestina* was assembled as a circular molecule of 124,153 bp with an overall GC content of 28.49% (Figure 1; Table S2). It exhibited the typical quadripartite structure of moss plastomes, consisting of a large single-copy (LSC) region of 85,756 bp, a small single-copy (SSC) region of 18,473 bp, and two inverted repeat (IR) regions of 9962 bp each.

A total of 126 genes, including duplicated IR copies, were annotated in the plastome. Based on functional classification, these genes were assigned to photosynthesis, self-replication, other functional genes, and genes of unknown function. The photosynthesis-related genes included genes encoding subunits of photosystem I, photosystem II, NADH dehydrogenase, cytochrome b/f complex, ATP synthase, rubisco, and protochlorophyllide reductase. The self-replication-related genes included ribosomal protein genes, RNA polymerase genes, rRNA genes, and tRNA genes. Other functional genes included *matK*, *clpP*, *cemA*, *accD*, and *infA*, whereas *ycf* genes were classified as conserved hypothetical chloroplast open reading frames (Table 1).

**Figure 2:**
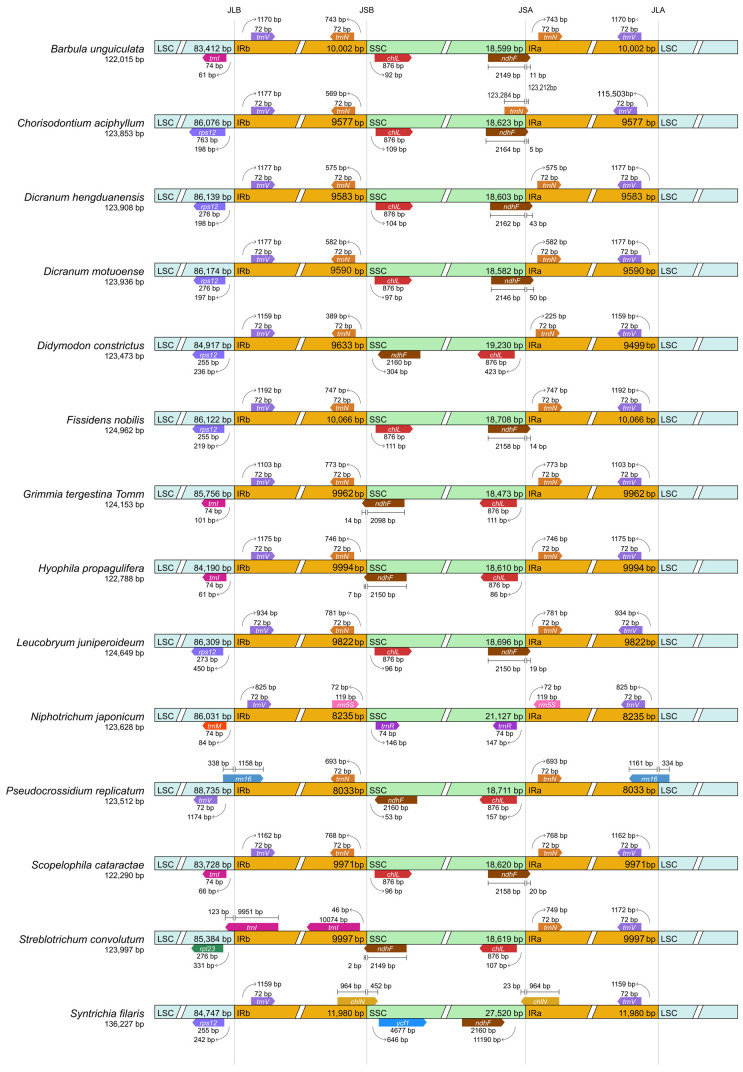
Comparison of LSC/IR/SSC junctions among *G. tergestina* and representative moss plastomes.

Nine genes were duplicated in the IR regions: *rrn16*, *rrn23*, *rrn4.5*, *rrn5*, *trnA-UGC*, *trnI-GAU*, *trnN-GUU*, *trnR-ACG*, and *trnV-GAC*. Fourteen genes contained introns, including three genes with two introns (*rps*12, *clp*P, and *ycf*3) and eleven genes with one intron. The detailed functional classification of annotated genes is provided in Table 1. The LSC/IR/SSC junctions of *G. tergestina* were generally comparable to those of representative moss plastomes, with no obvious large-scale structural rearrangement detected in the IR boundary comparison (Figure 2).

### 3.2. Repeat Composition and Codon Usage

A total of 569 cpSSRs were detected in the plastome of *G. tergestina*. Mononucleotide repeats were the most abundant class, accounting for 407 cpSSRs (71.53%), and were composed entirely of A/T motifs. Trinucleotide repeats were the second most frequent class (102), followed by dinucleotide repeats (32), tetranucleotide repeats (22), and pentanucleotide repeats (6). This A/T-rich SSR composition is consistent with the low GC content of the plastome.

Dispersed repeat analysis identified 222 repeats, including 68 forward repeats, 79 palindromic repeats, 51 reverse repeats, and 24 complement repeats. Most dispersed repeats were 30–40 bp in length. These repetitive elements provide a useful resource for future marker development but are not interpreted here as evidence of adaptive differentiation. Detailed cpSSR statistics are provided in Table S3, and dispersed repeat statistics are provided in Table S4.

RSCU analysis showed that 30 codons had RSCU values greater than 1. Among these, 22 codons ended with A or U, indicating a preference for A/U-ending codons (Figure S1). Given the AT-rich composition of the plastome, this pattern is best interpreted as a compositional feature rather than evidence of translational selection or lineage-specific adaptation. Supplementary ENC and PR2 analyses supported this conservative interpretation: most protein-coding genes were concentrated in the low-GC3s region of the ENC plot, and the PR2 plot showed an asymmetric distribution around the neutral point, indicating an imbalance among A, T, G, and C at the third codon position (Figure S2).

### 3.3. Nucleotide Diversity

**Figure 3:**
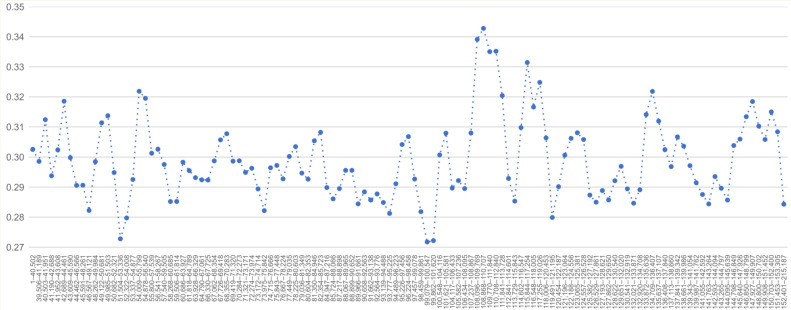
Sliding-window analysis of nucleotide diversity (π) among representative moss plastomes. Complete plastome sequences were aligned using MAFFT v7, and π values were calculated in DnaSP v6 using a window length of 1000 bp and a step size of 500 bp.

Sliding-window analysis of nucleotide diversity showed that most regions of the aligned plastome sequences were relatively conserved, whereas several windows exhibited comparatively higher π values (Figure 3). These regions may represent candidate variable regions for future marker development, although their utility should be further tested with broader sampling of Grimmiaceae and related mosses. Because the analysis was based on fixed-size windows across the complete plastome alignment, the observed peaks should be interpreted as regional nucleotide-diversity variation rather than as variation associated with individual short loci.

### 3.4. Phylogenetic Relationships

**Figure 4:**
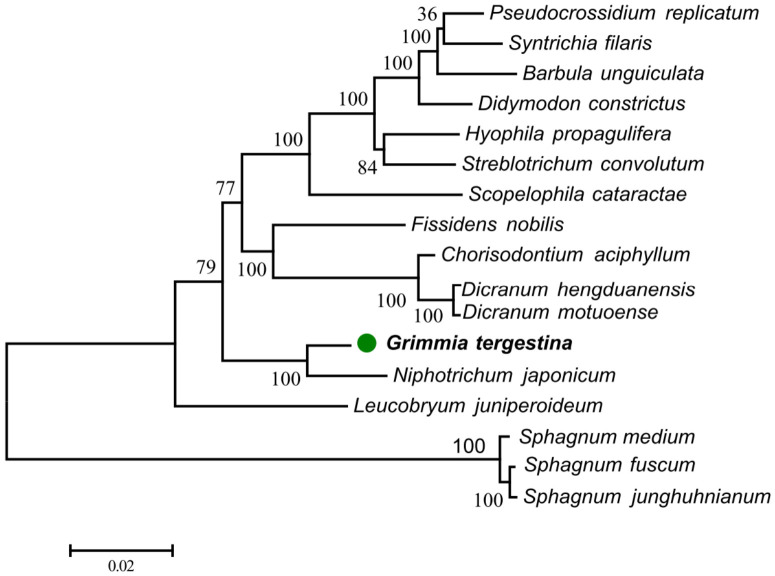
Maximum-likelihood phylogenetic tree inferred from shared chloroplast protein-coding genes of *G. tergestina* and representative moss plastomes. Numbers at nodes indicate bootstrap support values based on 1000 replicates. The green dot indicates *G. tergestina*. The tree provides preliminary placement of *G. tergestina* rather than comprehensive resolution of Grimmiaceae relationships.

The maximum-likelihood phylogenetic tree placed *G. tergestina* as sister to *N. japonicum* with strong bootstrap support (BS = 100; Figure 4). The two species formed a distinct clade within the sampled moss plastome dataset. The *Sphagnum* species were resolved as a separate outgroup clade, whereas the remaining moss taxa formed several subclades with variable bootstrap support.

## 4. Discussion

This Brief Report presents the first complete plastome of *G. tergestina* as a concise genomic resource for Grimmiaceae. The plastome length, gene content, GC content, and quadripartite organization are broadly consistent with previously reported moss plastomes, indicating that the chloroplast genome of *G. tergestina* is structurally conserved [23,24,25]. Similar patterns of conserved plastome organization have also been reported in other moss lineages, where plastome size variation is generally limited and is often associated with differences in non-coding regions, repeat composition, or IR boundary dynamics rather than major changes in core gene content [5,6,26]. Therefore, the plastome of *G. tergestina* is important as a baseline genomic resource, but it does not by itself indicate extensive plastome-level adaptation to lithophytic habitats. The IR boundary comparison also supported this structurally conservative interpretation, although denser sampling within Grimmiaceae will be required to evaluate lineage-specific junction variation.

The codon-usage and repeat analyses also support a conservative interpretation. The preference for A/U-ending codons is consistent with the AT-rich nucleotide composition of the plastome and with codon-usage patterns reported in other chloroplast genomes [27,28]. The supplementary ENC and PR2 analyses further support this conservative interpretation. Most genes were concentrated in the low-GC3s region of the ENC plot, and the PR2 plot showed an asymmetric distribution around the neutral point, indicating that codon usage is associated with nucleotide compositional bias and may also be affected by gene-specific constraints. However, because transcriptomic data and expression-based optimal codon analyses were not included, these results should not be interpreted as evidence of adaptive translational selection. The dominance of A/T-rich mononucleotide cpSSRs also reflects the same compositional bias. Chloroplast SSRs have been widely used as molecular markers in studies of plant genetic diversity, population structure, phylogeography, and evolutionary history [29,30]. However, the utility of these cpSSRs as population-level or species-discriminating markers cannot be inferred from a single plastome alone. Broader sampling across multiple accessions and related species will be required to evaluate their polymorphism, transferability, and discriminatory power [26,29,30]. The fixed-size sliding-window analysis further identified several relatively variable plastome regions, but these regions should be regarded only as preliminary candidates until their variability and discriminatory power are tested with denser taxon and population sampling.

The preliminary phylogenetic analysis placed *G. tergestina* in a clade with *N. japonicum*, consistent with its assignment to Grimmiaceae. Previous molecular studies of Grimmiaceae and *Grimmia* have shown that plastid markers are useful for resolving relationships within the family, but also that broader taxon sampling is necessary for stable inference of generic and species-level relationships [31,32]. The availability of chromosome-level genomic resources for *N. japonicum* further provides a useful reference point for future comparative studies within Grimmiaceae [33]. The plastome of *G. tergestina* is 525 bp longer than that of *N. japonicum* (124,153 bp versus 123,628 bp), corresponding to approximately 0.42% of the total plastome length. This small difference is more likely to reflect minor differences in non-coding regions or repeat composition than major changes in core gene content. Denser sampling of Grimmiaceae plastomes, especially from *Grimmia*, *Schistidium*, *Racomitrium*, *Bucklandiella*, and *Coscinodon*, will be necessary to test this inference rigorously.

Several limitations remain. First, this study reports a single newly sequenced plastome and therefore should not be used to make broad claims about lithophytic adaptation. Second, the phylogenetic analysis is limited by the availability of complete plastomes from Grimmiaceae and related mosses. Third, organellar genomes represent only one component of the evolutionary history of a species, and plastome-based patterns may not fully reflect nuclear genomic history, population processes, or ecological diversification [34]. Future studies integrating plastome, mitogenome, nuclear genomic, ecological, and population-level data will be needed to explore evolutionary diversification in *Grimmia* and related lithophytic mosses.

## 5. Conclusions

The complete plastome of *G. tergestina* was assembled and characterized for the first time. The plastome shows a conserved quadripartite structure, typical moss plastome gene content, A/T-rich sequence composition, and abundant chloroplast repeats. Codon-usage analyses and nucleotide diversity analysis provide additional descriptive information for future marker development, but these results should be validated with broader taxon and population sampling. Preliminary phylogenetic analysis supports the placement of *G. tergestina* within Grimmiaceae. This newly reported plastome enriches genomic resources for *Grimmia* and provides a useful foundation for future studies of species identification, phylogeny, and comparative plastome evolution in Grimmiaceae.

## Figures and Tables

**Table 1:** Functional classification of genes in the chloroplast genome of *Grimmia tergestina*.

Category	Gene Group	Gene Name
Photosynthesis	Subunits of photosystem I	*psaA*, *psaB*, *psaC*, *psaI*, *psaJ*, *psaM*
	Subunits of photosystem II	*psbA*, *psbB*, *psbC*, *psbD*, *psbE*, *psbF*, *psbH*, *psbI*, *psbJ*, *psbK*, *psbL*, *psbM*, *psbN*, *psbT*, *psbZ*
	Subunits of NADH dehydrogenase	ndhA*, ndhB*, *ndhC*, *ndhD*, *ndhE*, *ndhF*, *ndhG*, *ndhH*, *ndhI*, *ndhJ*, *ndhK*
	Subunits of cytochrome b/f complex	*petA*, *petB*, *petD*, *petG*, *petL*
	Subunits of ATP synthase	*atpA*, *atpB*, *atpE*, atpF*, *atpH*, *atpI*
	Large subunit of rubisco	*rbcL*
	Subunits of protochlorophyllide reductase	*chlB*, *chlL*, *chlN*
Self-replication	Proteins of large ribosomal subunit	*rpl14*, *rpl16*, rpl2*, *rpl20*, *rpl21*, *rpl22*, *rpl23*, *rpl32*, *rpl33*, *rpl36*
	Proteins of small ribosomal subunit	*rps11*, rps12**, *rps14*, *rps15*, *rps18*, *rps19*, *rps2*, *rps3*, *rps4*, *rps7*, *rps8*
	Subunits of RNA polymerase	*rpoB*, rpoC1*, *rpoC2*
	Ribosomal RNAs	*rrn16(2)*, *rrn23(2)*, *rrn4.5(2)*, *rrn5(2)*
	Transfer RNAs	trnA-UGC*(2), *trnC-GCA*, *trnD-GUC*, *trnE-UUC*, *trnF-GAA*, *trnG-UCC*, *trnH-GUG*, *trnI-CAU*, trnI-GAU*(2), trnK-UUU*, *trnL-CAA*, trnL-UAA*, *trnL-UAG*, *trnM-CAU*, *trnN-GUU(2)*, *trnP-GGG*, *trnP-UGG*, *trnQ-UUG*, *trnR-ACG(2)*, *trnR-CCG*, *trnR-UCU*, *trnS-GCU*, *trnS-GGA*, *trnS-UGA*, *trnT-GGU*, *trnT-UGU*, *trnV-GAC(2)*, trnV-UAC*, *trnW-CCA*, *trnY-GUA*, *trnfM-CAU*
Other genes	Maturase	*matK*
	Protease	clpP**
	Envelope membrane protein	*cemA*
	Acetyl-CoA carboxylase	*accD*
	Translation initiation factor	*infA*
Genes of unknown function	Conserved hypothetical chloroplast ORF	*ycf1*, *ycf12*, *ycf2*, ycf3**, *ycf4*, ycf66*

Notes: Gene* indicates a gene with one intron; Gene** indicates a gene with two introns; Gene(2) indicates the copy number of multicopy genes.

## Data Availability

The data presented in this study are openly available in the China National GeneBank at https://www.cngb.org (accessed on 24 November 2025) under accession number CNS1408476, and in GenBank under accession number PZ382285.

## Supplementary Materials

The following supporting information can be downloaded at https://www.mdpi.com/article/10.3390/genes17050572/s1, Table S1, species included in the phylogenetic analysis and their chloroplast genome accessions; Table S2, basic features, sequencing, and assembly statistics of the plastome of *G. tergestina*; Table S3, distribution of chloroplast simple sequence repeats (cpSSRs) in the plastome of *G. tergestina*; Table S4, distribution of dispersed repeats in the plastome of *G. tergestina*; Figure S1, RSCU results of chloroplast protein-coding genes; Figure S2, ENC plot, PR2 plot, and gene-wise ENC distribution of chloroplast protein-coding genes.
