# Supplementary material for: Complete Plastome Sequence of Grimmia tergestina Provides a Genomic Resource for Grimmiaceae
Source: Genes (Basel). 2026 May 18;17(5):572. doi: 10.3390/genes17050572 (PMC13205997; doi:10.3390/genes17050572)
Supplement: Supplementary file 1 [file genes-17-00572-s001.zip › genes-4258594-supplementary.pdf]

Table S1. Species included in the phylogenetic analysis and their chloroplast genome accessions.

Subclass	Order	Family	Genus	Species	Accession No.	Genome Size
Sphagnopsida	Sphagnales	Sphagnaceae	*Sphagnum*	*Sphagnum fuscum*	PP820908.1	139,046 bp
Sphagnopsida	Sphagnales	Sphagnaceae	*Sphagnum*	*Sphagnum junghuhnianum*	OK105067.1	138,947 bp
Sphagnopsida	Sphagnales	Sphagnaceae	*Sphagnum*	*Sphagnum medium*	PP820907.1	140,336 bp
Dicranidae	Grimmiales	Grimmiaceae	*Grimmia*	*Grimmia tergestina*	CNS1408476/ PZ382285	124,153 bp
Dicranidae	Pottiales	Pottiaceae	*Barbula*	*Barbula unguiculata*	LC747006.1	122,015 bp
Dicranidae	Dicranales	Dicranaceae	*Chorisodontium*	*Chorisodontium aciphyllum*	MW355440.1	123,853 bp
Dicranidae	Dicranales	Dicranaceae	*Dicranum*	*Dicranum hengduanensis*	OQ401774.1	123,908 bp
Dicranidae	Dicranales	Dicranaceae	*Dicranum*	*Dicranum motuoense*	PQ821713.1	123,936 bp
Dicranidae	Pottiales	Pottiaceae	*Didymodon*	*Didymodon constrictus*	ON310498.1	123,473 bp
Dicranidae	Dicranales	Fissidentaceae	*Fissidens*	*Fissidens nobilis*	MK876184.1	124,962 bp
Dicranidae	Pottiales	Pottiaceae	*Hyophila*	*Hyophila propagulifera*	LC747008.1	122,788 bp
Dicranidae	Dicranales	Leucobryaceae	*Leucobryum*	*Leucobryum juniperoideum*	MK952779.1	124,649 bp
Dicranidae	Grimmiales	Grimmiaceae	*Niphotrichum*	*Niphotrichum japonicum*	OR777684.1	123,628 bp
Dicranidae	Pottiales	Pottiaceae	*Pseudocrossidium*	*Pseudocrossidium replicatum*	MG132071.1	123,512 bp
Dicranidae	Pottiales	Pottiaceae	*Scopelophila*	*Scopelophila cataractae*	LC634773.1	122,290 bp
Dicranidae	Pottiales	Pottiaceae	*Streblotrichum*	*Streblotrichum convolutum*	LC747010.1	123,997 bp
Dicranidae	Pottiales	Pottiaceae	*Syntrichia*	*Syntrichia filaris*	MK852705.1	136,227 bp

Table S2. Basic features, sequencing, and assembly statistics of the plastome.

Feature	Value
Genome size	124,153 bp

Genome structure	Quadripartite plastome with LSC, SSC, and two IR regions
LSC length	85,756 bp
SSC length	18,473 bp
IR length	9,962 bp each
Overall GC content	28.49%
Total annotated genes	126
Protein-coding genes	82
tRNA genes	36
rRNA genes	8
Duplicated IR genes	9
Intron-containing genes	14
cpSSRs	569
Dispersed repeats	222
CNGB accession	CNS1408476
GenBank accession	PZ382285

Table S3. Distribution of chloroplast SSRs in *Grimmia tergestina*.

SSR Type	Repeat Motif	Count
Mononucleotide	A/T	407
Dinucleotide	AT/AT	31
Dinucleotide	AG/CT	1
Trinucleotide	AAC/GTT	3
Trinucleotide	AAG/CTT	7
Trinucleotide	AAT/ATT	80
Trinucleotide	ACC/GGT	1
Trinucleotide	ACG/CGT	1
Trinucleotide	ACT/AGT	1
Trinucleotide	AGC/CTG	3
Trinucleotide	AGG/CCT	1
Trinucleotide	ATC/ATG	5
Tetranucleotide	AAAC/GTTT	2
Tetranucleotide	AAAT/ATTT	18
Tetranucleotide	ACCT/AGGT	2
Pentanucleotide	AAAAG/CTTTT	1
Pentanucleotide	AAAAT/ATTTT	4
Pentanucleotide	AATAG/ATTCT	1

Table S4. Distribution of dispersed repeats in the chloroplast genome of *Grimmia tergestina*.

Length (bp)	Repeat Type	Number
30–39	P	60
30–39	F	66
30–39	R	50
30–39	C	23

40–49	P	6
40–49	F	0
40–49	R	1
40–49	C	1
50–59	P	3
50–59	F	1
50–59	R	0
50–59	C	0
60–69	P	5
60–69	F	1
60–69	R	0
60–69	C	0
70–79	P	1
70–79	F	0
70–79	R	0
70–79	C	0
90–160	P	3
90–160	F	0
90–160	R	0
90–160	C	0
≥200	P	1
≥200	F	0
≥200	R	0
≥200	C	0

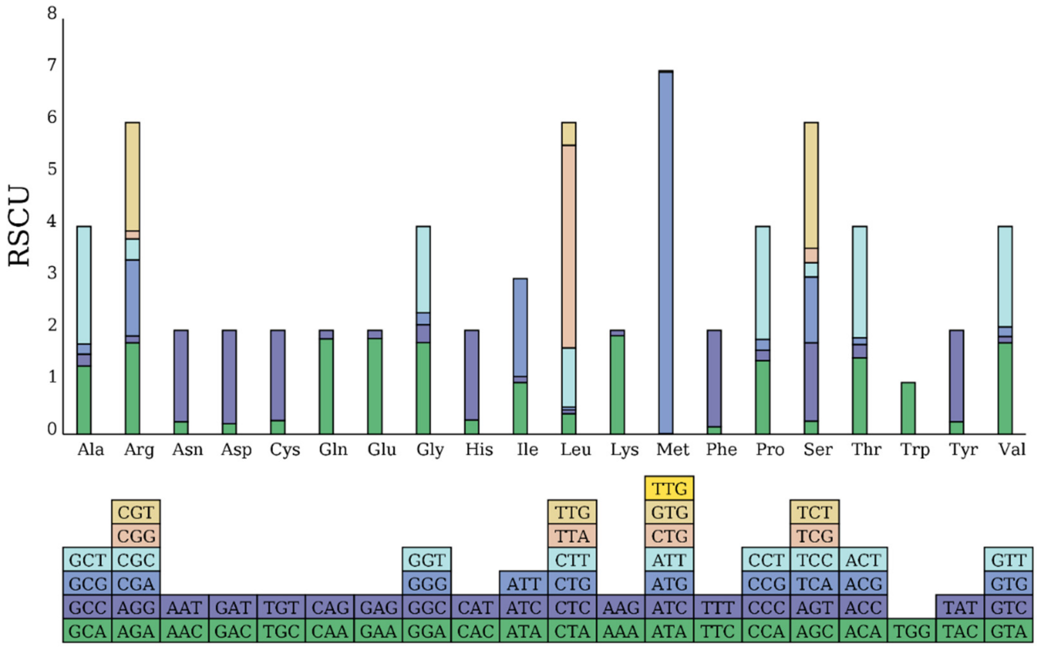

Figure S1. Histogram of relative synonymous codon usage (RSCU) in the chloroplast genome of *Grimmia tergestina*.

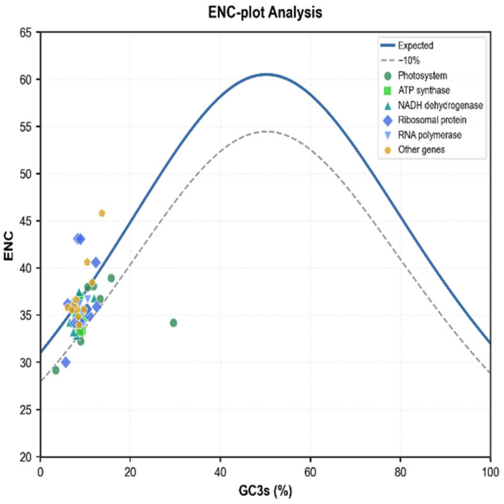

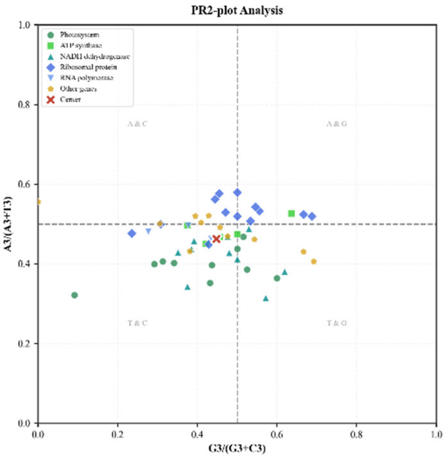

(a) (b)

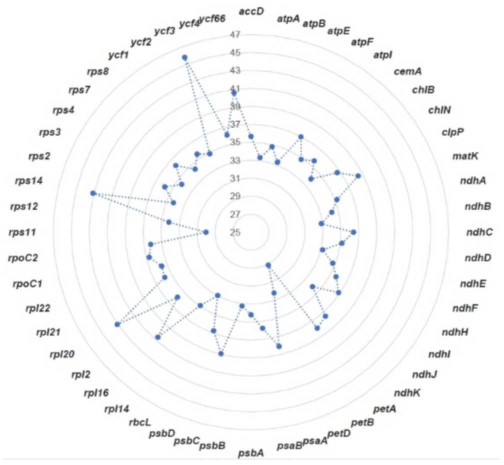

(c)

Figure S2. Supplementary codon-usage analyses of chloroplast protein-coding genes in *Grimmia tergestina*. (**a**) ENC plot showing the relationship between effective number of codons (ENC) and GC content at the third synonymous codon position (GC3s). The solid curve represents the expected ENC values under compositional constraint, and the dashed curve represents 10% below the expected curve. (**b**) PR2 plot showing the relationships between G3/(G3 + C3) and A3/(A3 + T3) at the third codon position. Dashed lines indicate the neutral point of 0.5. (**c**) Gene-wise ENC distribution among chloroplast protein-coding genes. Gene functional categories are indicated by different symbols or colors.
